# Early maternal depressive symptoms and child growth trajectories: a longitudinal analysis of a nationally representative US birth cohort

**DOI:** 10.1186/1471-2431-14-185

**Published:** 2014-07-21

**Authors:** Pamela J Surkan, Anna K Ettinger, Rebecca S Hock, Saifuddin Ahmed, Donna M Strobino, Cynthia S Minkovitz

**Affiliations:** 1 Social and Behavioral Interventions Program, Department of International Health, Johns Hopkins Bloomberg School of Public Health, 615 North Wolfe St., Room E5523, Baltimore, MD 21205-2179, USA; 2 Department of Population, Family and Reproductive Health, Johns Hopkins Bloomberg School of Public Health, Baltimore, MD, USA; 3 Division of Global Psychiatry Massachusetts General Hospital, Harvard Medical School, Boston, MA, USA

**Keywords:** Height, Body mass index, Child growth, Longitudinal, Postpartum depression

## Abstract

**Background:**

Maternal depressive symptoms are negatively associated with early child growth in developing countries; however, few studies have examined this relation in developed countries or used a longitudinal design with data past the second year of the child’s life. We investigated if and when early maternal depressive symptoms affect average growth in young children up to age 6 in a nationally representative sample of US children.

**Methods:**

Using data from 6,550 singleton births from the Early Childhood Longitudinal Study -- Birth Cohort (ECLS-B), we fit growth trajectory models with random effects to examine the relation between maternal depressive symptoms at 9 months based on the twelve-item version of the Center for Epidemiologic Studies Depression Scale (CES-D) and child height and body mass index (BMI) to age 6 years.

**Results:**

Mothers with moderate/severe depressive symptoms at 9 months postpartum had children with shorter stature at this same point in time [average 0.26 cm shorter; 95% CI: 5 cm, 48 cm] than mothers without depressive symptoms; children whose mothers reported postpartum depressive symptoms remained significantly shorter throughout the child’s first 6 years.

**Conclusions:**

Results suggest that the first year postpartum is a critical window for addressing maternal depressive symptoms in order to optimize child growth. Future studies should investigate the role of caregiving and feeding practices as potential mechanisms linking maternal depressive symptoms and child growth trajectories.

## Background

Postpartum depressive symptoms are common, with an estimated US prevalence of 10-15% [1], and are associated with impaired parenting practices and non-responsive feeding practices [2,3]. A recent meta-analysis of studies from developing countries showed an effect of maternal depressive symptoms on both underweight and stunting [4]. Maternal symptoms have also been related to child overweight and higher body mass index (BMI) in some studies [5,6], but not in others [7-9]. Both under and over-nutrition in children may lead to long-term negative social and health consequences [10,11].

Longitudinal growth research using diverse samples has been mostly limited to the first two years of life and has shown mixed result [4,12]. Our prior research indicated that maternal depressive symptoms were associated with increased odds of stature below the 10th percentile when children were ages 4 and 5 years old [13]. Nevertheless, the timing of onset of differences in children with and without depressive symptoms is not known. Moreover, due to accelerated growth and potential variations in growth patterns in the first year of life [14], understanding the way in which early maternal depressive symptoms affects growth trajectories in the interceding years may inform intervention efforts. Due to catch-up growth, early growth deficits or delays may be transient rather than long lasting. Alternatively, some research suggests that early under-nutrition and growth faltering can continue over time [15]. We studied whether the influence of early maternal depressive symptoms persists between nine months and 6 years of age and whether this influence varied by age. Existing literature suggests that early maternal depressive symptoms affect parenting, including feeding practices [2,3]. In addition, given evidence that parental feeding practices and eating behaviors are established in early childhood [16-18], we hypothesized that the consequences of early parenting practices related to maternal depressive symptoms may result in lasting sequelae. In particular, we examined if depressive symptoms at 9 months postpartum were related to children’s height and BMI trajectories through age 6 in a nationally representative sample of children from the United States.

Given changing growth rates during early childhood, our study fills a gap in understanding how the effects of maternal depressive symptoms on child growth may vary by age. This study extends our previous work [13] by using growth curve modeling with random effects to investigate the impact of maternal depressive symptoms on child growth trajectories, while allowing for individual variability in growth patterns of height and BMI [19].

## Methods

**Figure 1:**
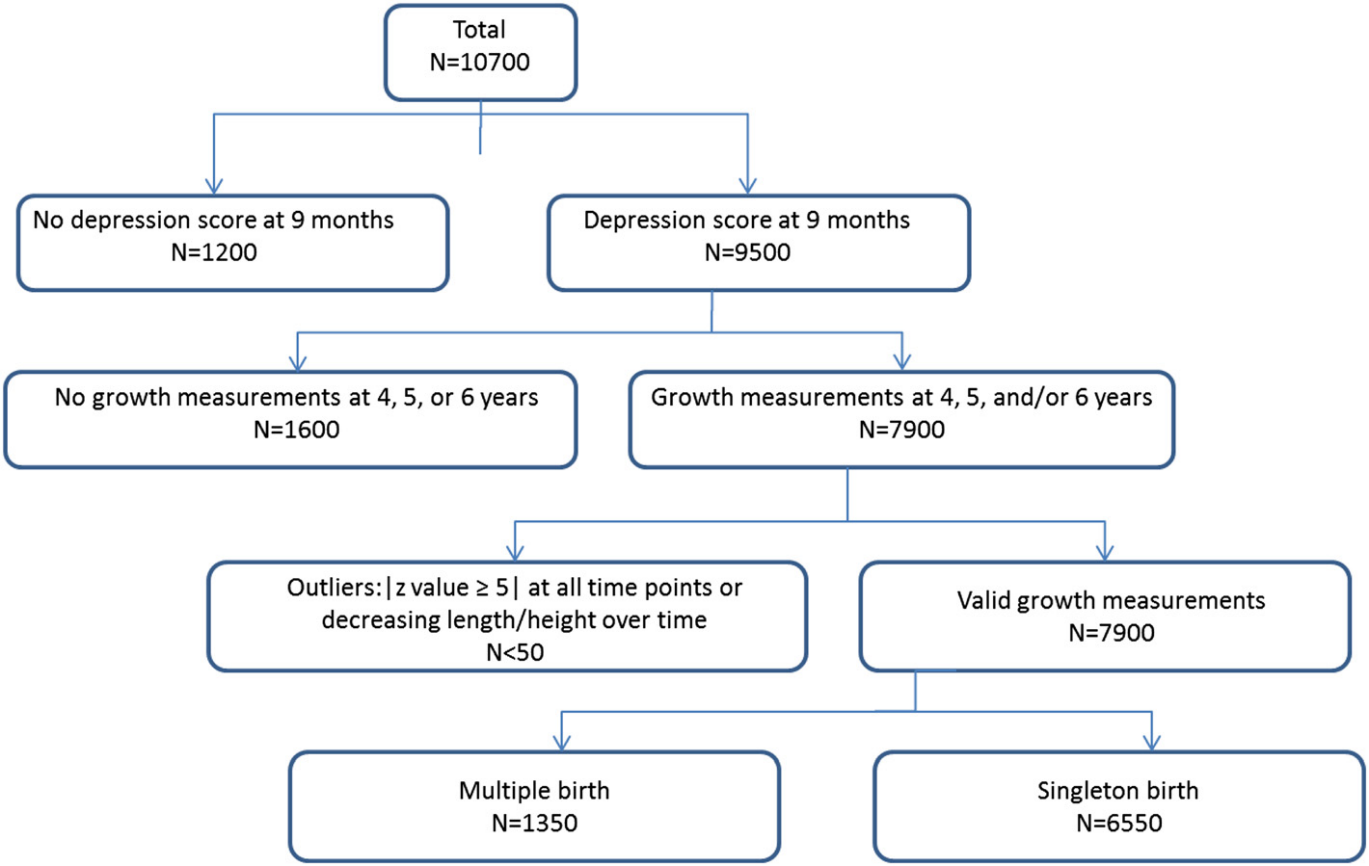
Exclusion criteria on left side of flowchart and inclusion criteria on right side, leading to selection of study participants (N = 6550).

We used data from the Early Childhood Longitudinal Study – Birth Cohort (ECLS-B), a prospective, longitudinal study of a nationally representative sample of approximately 10,700 children born in the US in 2001 and followed through kindergarten. The ECLS-B was conducted by the US Department of Education Institute of Education Sciences National Center for Education Statistics (NCES) in collaboration with several other federal agencies. Multiple births, low birth weight, and selected ethnic minority children, including American Indians, were oversampled. Children born to mothers less than 15 years old and infants who died or were adopted before 9 months were excluded. Our analyses included data from birth certificates, and from the 9 month, 4 year (preschool sample), 5 year (2006–2007 kindergarten sample), and 6 year (2007–2008 kindergarten sample) waves of data collection. Data included direct child assessments during home visits, parent/caregiver computer assisted personal interviews (CAPI), self-administered questionnaires at 9 months, and audio-computer assisted parent (or other caregiver) interviews at 4, 5, and 6 years for sensitive items. The weighted CAPI response rates ranged between 54-74% [20], and weighted child assessment response rates for children with parental data ranged between 96-99% across time points [21-23]. The majority of children (~72%) were followed to 5 years when they entered kindergarten. Children who were not age-eligible to enter kindergarten in 2006 were also included in the 2007 kindergarten sample (n = 1,300), along with a small percentage of children (~5%) who repeated kindergarten. We used all available measurements on child height and weight.Our sample included approximately 6,550 children whose mothers reported data about depressive symptoms at 9 months. Children included in the height trajectory analyses had at least two valid height measurements, and those included in the BMI analysis at least two valid BMI values. Multiple births (n = 1,350) were excluded because of potentially different growth trajectories than singletons. We examined weight trajectories over time and changes in weight between time points for implausible values and outliers (more than 3 standard deviations (SD) above average weight gain for two time points). We also examined the effect of height and BMI outliers on our estimates; exclusion of outliers (>3 SD or < −3 SD for height and BMI) did not change the parameters so our final sample included these observations. In the final sample, approximately 6,000 children had valid measures at 4 years, 4,600 at 5 years, and 1,300 at 6 years. For a flow diagram of participants included in and excluded from the study, please see Figure 1.

Maternal depressive symptoms were assessed using a twelve-item version of the Center for Epidemiological Studies Depression Scale (CES-D) [24] administered at 9 months. The CES-D assesses depressive symptoms during the past week using a four-point Likert scale: 0 = rarely or never, 1 = some or a little, 2 = occasionally or moderately, and 3 = most or all [25]. The twelve-item scale yields a total score from 0–36, which we categorized into three groups: scores <5 (no symptoms), 5–9 (mild symptoms), and ≥10 (moderate to severe symptoms). The moderate to severe symptoms score of ≥10 corresponds to the score of ≥16 used as the standard cutoff for a high level of depressive symptoms on the original 20-item scale. The twelve-item version CES-D has been validated and applied in other large national studies [26]. Internal consistency for the CES-D short form in our sample was high (Cronbach ***α*** = 0.88).

We omitted mothers who were missing 9 or more of the 12 items from the CES-D scale (n = 1200), but respondents with at least four completed items were included in the analyses (~9,500 mothers, 88.9% of the original sample.) The majority of mothers completed all items (n = 6,150) or were missing only one (n = 250); less than 150 mothers were missing two or more items. Scores for missing items were imputed using the average scale score from the completed items. Sensitivity analyses using a more stringent cut-off for CES-D completed items (only including mothers missing 0 or 1 items) did not yield significant differences in the model estimates (data not shown).

Child length/height and BMI were directly measured by trained interviewers at home visits at each wave of data collection. At 9 months, a Measure Mat was used to assess children’s recumbent length. At ages 4–6 years, children’s standing height was assessed using a Portable Stadiometer. Children’s weight at 9 months was assessed by first weighing the mother and child together on a digital scale; the mother’s weight was then subtracted to obtain the child’s weight [27]. At later ages, children were weighed independently [25]. At 9 months, two measurements were averaged for weight and length scores. If the difference between the two measures exceeded 5%, the weight or height measurement closest to the weighted average for sample children of the same age and birth weight was used [27]. Likewise, at 4 years, two measurements were taken and averaged unless measurement differences exceeded 5%, in which case the field interviewer checked the measurements to confirm that no error had occurred and re-measured the child if necessary [20]. At ages 5 and 6, three measurements were taken for each assessment and the closest two measurements were averaged [20].

BMI was calculated as weight (kg) divided by height (m) squared for children 4–6 years of age. BMI was not calculated for 9 month old children because it is not typically used for children under 2 years. Since BMI is not a preferred nutritional indicator in children under 2, we also used weight-for-length z-score at 9 months along with BMI z-scores at years 4, 5 and 6 based on the US 2000 Growth Charts (using the zanthro command in Stata). We also examined weight-for-length (at 9 months) and weight-for-height (4–6 years) z-scores at each time point.

Analyses adjusted for socio-demographic, household, maternal and child characteristics based on maternal report at 9 months unless otherwise noted. Household income was categorized as: <$25,000, $25,000-$49,999, $50,000-$99,999, and ≥ $100,000. Household food security was measured by 18 items from the US Department of Agriculture Household Food Security Scale, assessing food availability and hunger over the past twelve months [28]. It was categorized as secure or insecure; food-insecure households included those with and without hunger. Home ownership was measured as a dichotomous variable (owned or not owned home) as was family structure (single or two-parent family).

Maternal characteristics included age, race/ethnicity (Non-Hispanic (NH) White, NH Black, NH Asian, Hispanic, and Other), education (some High School (HS) or less, HS graduate, some college, college and beyond), prepregnancy weight, weight gain during pregnancy (without subtracting birth weight), parity, and smoking status. Child characteristics included child sex, birth weight and gestational age (from birth certificate), age at interview, overall child health status, and whether the child was ever breastfed.

We used multiple imputation to impute missing values for covariates with missing responses using 10 imputed datasets; all covariates were missing less than 3% of responses. Sensitivity analyses were also conducted using complete case analyses and nearest neighbor hot deck analyses, which produced similar results.

### Statistical analysis

We first evaluated the association between maternal depressive symptoms and the covariates using chi-square statistics, and unadjusted logistic regression. Exploratory data analyses of child height and BMI over time included graphing cross-sectional box plots, average trajectories, and individual spaghetti plots to examine the shape of trajectories and identify potential outlying values. Unadjusted longitudinal analyses of the associations between height and BMI and all covariates were conducted using random effects models, including random intercepts and slopes. Covariates measured at baseline were selected based on significant unadjusted associations (*P* <0.05) with height or BMI or because they were conceptually related to child growth. Assessment of time-varying covariates with height and BMI trajectories did not produce meaningful differences in the model estimates; baseline covariates were used in the final models.

Non-linearity of growth models was accounted for by including quadratic terms for child age in the final models for both height and BMI. Both partially adjusted (controlling for child age and sex) and fully adjusted models (controlling for all covariates) were fit. Main effect models examined a linear shift in depressive symptoms (main effect models). Interaction models included an interaction term for maternal depressive symptoms and child age to determine if growth trajectories varied by the levels of maternal depressive symptoms over time. The analysis resulted in four models each for child height and BMI trajectories: Model 1a, c) partially adjusted main effect models; Model 1b, d) fully adjusted main effect models; Model 2a, c) partially adjusted interaction models; and 2b, d.) fully adjusted interaction models. We conducted two additional analyses using 1.) combined weight-for-length z-scores (9 months) and BMI z-scores (4–6 years) and 2) weight-for-length/height z-scores at all ages. The models using BMI values (kg/m2) showed similar results to the BMI trajectory models with z-scores, so we present the results for the BMI trajectory models using BMI values due to their clinical relevance and ease of interpretation. Model fit was assessed by comparing the Akaike information criterion (AIC) values of potential models and conducting likelihood ratio tests of nested models (main effects vs. growth trajectory models for height and BMI).

Random effects were used to examine the relation of maternal depressive symptoms with child height from age 9 months to 6 years and BMI from age 4 to 6 years, adjusting for socio-demographic and child health covariates. Since coefficient and standard error estimates did not change substantially between the models using independent, exchangeable, and unstructured covariance between the random slopes and intercepts, independent covariance was specified in the final models for simplicity. Analyses were conducted using Stata 11.0 (Statacorp, College Station Texas). *P*-values were based on two-sided tests.

Weighted analyses were conducted for descriptive statistics and point estimates due to oversampling of particular groups. Unweighted analyses were performed for regression models because the analyses focused on the relations between variables rather than prevalence or point estimates [29].

The ECLS-B is a restricted-use secondary dataset. The authors received a restricted-use license and access to the data from the US Department of Education National Center for Education Statistics (NCES). This study was approved by the NCES and Johns Hopkins Bloomberg School of Public Health Institutional Review Board for human subject research. The authors abided by the confidentiality regulations and restrictions for using the data and rounded all figures to the nearest 50 based on the NCES data reporting requirements. This manuscript was submitted to NCES for a disclosure review and was approved for publication.

## Results

The sample included 6,550 children, of whom 57% were non-Hispanic White, 13% non-Hispanic Black, 23% Hispanic children, and the remainder, another race/ethnicity (weighted estimates; see Additional file 1). At 9 months, the majority of the respondents were the biological mothers (99%). Most households had two parents (81%) and were food secure (89%). The socioeconomic characteristics of families were variable; 35% of households had incomes below $25,000 per year, while 11% had incomes above $100,000. Only 18 percent of mothers had less than a high school education, with 27% having completed college or more (See Surkan et al. 2012 for details on other socio-demographic variables [13]). Children who were dropped from the study due to missing data were more likely to be racial and ethnic minorities and to have poor health status. Mothers excluded from analyses due to missing data had lower incomes, were less likely to own their homes, to be working, and to have food secure households, and more likely to be younger, racial/ethnic minorities, smokers, less educated, and single parents than study mothers (data available upon request; *P* < 0.05 for the differences reported above).

At 9 months, weighted estimates indicated that 66% of mothers had no depressive symptoms, 19% had mild symptoms, and 14% had moderate/severe symptoms (data not shown). Mothers who were single, young, and in low socio-economic (including income, food security, and home ownership) and education categories had increased prevalence of moderate/severe depressive symptoms than mothers without these characteristics at 9 months.

In both partially (controlling for only child age and sex) and fully adjusted (controlling for all covariates) analyses, children whose mothers had moderate to severe depressive symptoms at 9 months remained shorter over time than the reference population (partially adjusted β = −0.45 [95% Confidence Interval (CI):-0.69,-0.30]; fully adjusted β = −0.26 [95% CI:-0.48,-0.05] based on Models 1 a and b: Main effect models (Table 1). The interaction terms between depressive symptoms and child age were not significant (Table 1). Adding the estimates for the interaction between depressive symptoms and child age to the coefficients of the main effects showed no significant change in the effect of moderate/severe maternal depressive symptoms on child height (data not shown). A goodness-of-fit test (likelihood ratio test) comparing the log-likelihood ratios between the two models also indicated that the interaction model did not significantly improve model fit compared to the main effect model (p = 0.52), suggesting that the difference in stature between children of mothers with and without moderate/severe depressive symptoms remained constant during the child’s first 6 years of life.

**Table 1:** Longitudinal models of maternal depressive symptoms on child height and BMI between 9 months and 6 years

	**Height (cm)**		**BMI (kg/m**^**2**^**)**	
	**a. Partially adjusted**^**1**^	**b. Fully adjusted**^**2**^	**c. Partially adjusted**^**1**^	**d. Fully adjusted**^**2**^
	**β (95% CI)**	**β (95% CI)**	**β (95% CI)**	**β (95% CI)**
Model 1: Main effects models (Linear shift in maternal depression)				
Maternal depressive symptoms				
None	Reference			
Mild	−0.20 (−0.41, 0.008)^	−0.08 (−0.27, 0.11)	0.14 (0.02, 0.26)*	0.10 (−0.01, 0.22)
Moderate/severe	−0.43 (−0.66, −0.19)***	−0.26 (−0.48, −0.05)*	0.08 (−0.05, 0.21)	−0.02 (−0.16, 0.11)
Model 2: Interaction models (Interactions of maternal depressive symptoms and child age terms)				
Maternal depressive symptoms				
None	Reference			
Mild	−0.22 (−0.44, 0.005)^	−0.11 (−0.31, 0.09)	−0.07 (−0.39, 0.26)	−0.09 (−0.42, 0.25)
Moderate/severe	−0.45 (−0.69, −0.30)***	−0.23 (−0.46, −0.50)*	0.17 (−0.19, 0.53)	0.05 (−0.32, 0.42)
Maternal symptoms x child age_ij_				
None	Reference			
Mild	0.02 (−0.16, 0.20)	0.002 (−0.01, 002)	0.13 (−0.12, 0.37)	0.01 (−0.01, 0.03)
Moderate/severe	−0.01 (−0.21, 0.19)	−0.003 (−0.02, 0.01)	−0.05 (−0.32, 0.22)	−0.002 (−0.03, 0.02)
Maternal symptoms x child age_ij_^2^				
None	Reference			
Mild	−0.002 (−0.04, 0.04)	<−0.0001 (−0.0003, 0.0003)	−0.01 (−0.05, 0.03)	−0.0001 (−0.0004, 0.0002)
Moderate/severe	0.01 (−0.03, 0.06)	0.0001 (−0.0002, 0.0004)	0.005 (−0.04, 0.05)	0.00002 (−0.0003, 0.0003)

Beta coefficients reported in this analysis have not been standardized.

^1^Random effects (intercept + slope) models adjusted for child sex and age (entered at 9 months).

^2^Random effects (intercept + slope) model adjusted for child sex and age (centered at 9 months) and baseline characteristics: maternal age, education, employment, home ownership, race, pre-pregnancy weight, pregnancy weight gain, smoking status, and child health status, ever breast fed, gestational age, and birthweight.

*p-value <0.05; **p-value <0.01; ***p-value <0.001.

^p-value <0.10.

In the partially adjusted main effects model for child BMI (Model 1c), children of mothers with mild depressive symptoms at 9 months on average had higher BMIs (β = 0.14, [95% CI: 0.02, 0.26], Table 1) than children of mothers without symptoms. Both partially adjusted and fully adjusted interaction models, however, showed no significant variation in BMI during early childhood by mild or moderate/severe depressive symptoms (Table 1). The interaction model (Model 2d) did not fit the data better than the main effects model (Model 1d) for BMI (Likelihood ratio test, p = 0.27). Likewise, there were no significant effects of different levels of maternal depressive symptoms on models combining child weight-for-length and BMI z-scores in trajectories or trajectories of weight-for-length z-scores (data not shown).

## Discussion

Our longitudinal growth trajectory analyses suggest that, although the effects were modest, children of mothers with greater levels of depressive symptoms during the postpartum period (at 9 months) had lower attained height beginning at 9 months that persisted to age 6 compared to children of mothers with no depressive symptoms. Our previously reported findings showed that maternal depressive symptoms were associated with increased odds of stature below the 10th percentile at later points in time (4 or 5 years) [13]. The results of our current study more fully describe the relation between maternal depressive symptoms and infant growth by demonstrating that the association between maternal symptoms and decreased child stature is established at 9 months, and the difference remains constant over the child’s first 6 years of life. The persistent association of maternal depressive symptoms during the first year with height at 6 years of age is notable, as few studies to date have follow-up as late as kindergarten.

Consistent with our findings, most literature in developing countries has shown maternal depressive symptoms to be related to growth deficits [4], although these studies have not undertaken trajectory analyses. In contrast to our results, Ertel et al. found that postpartum depressive symptoms predicted taller rather than shorter child stature in children between ages six months to three years in a longitudinal study of a primarily white, affluent US sample with health insurance [30]. Our findings are based on a nationally representative sample of US singleton children, which is more variable in terms of SES than the affluent sample studied by Ertel and colleagues. We also used a different instrument to measure depressive symptoms.

The difference in height between the children of mothers with and without moderate and severe symptoms was modest. Nonetheless, these findings have important clinical and public health implications since maternal depressive symptoms are modifiable and child stature is a key indicator of long-term nutrition and health status [10]. The results reinforce the importance of preventing maternal depressive symptoms early in life to help place children on optimal height trajectories.

As child obesity is a growing public health concern in the US, the present analyses also examined BMI trajectories. We found an association between mild maternal depressive symptoms and child BMI over time, but no relation between moderate to severe depressive symptoms and child BMI. This finding adds to mixed results of studies in both developed and developing countries, showing positive, negative, and null associations between maternal depressive symptoms and child BMI [4,7,8,30]. One study showed an association between maternal postpartum depressive symptoms and child overall adiposity using data from birth to age three, but no relation was observed with BMI z-score, weight-for-height z-score or the ratio of subscapular to tricep skinfold (a measure of central adiposity) [30]. A multi-center study, including children in Belgium, Italy, Spain, Poland and Germany, showed lower weight-for-length z-scores for children of mothers with higher symptoms of depression at age two, but no relation with BMI or other anthropometric indicators [9]. In a study of Latina mothers, children of women who had depressive symptoms both prenatally and at 4–6 weeks postpartum were more likely to be underweight, have less weight gain, and less likely to be overweight (>85% of weight-for-length) between 6 months and two years of age [31]. Another US study showed that maternal depressive symptoms measured repeatedly at 1, 24 or 36 months postpartum, predicted child overweight in grade school [32]. Given inconsistencies in the literature and the weak association between mild depressive symptoms and child BMI in our study, this finding could be due to chance. Further research is needed to confirm this association.

Mechanisms explaining the observed relations are unclear, although caregiving practices have been suggested. Non-responsive feeding in younger children is characterized by lack of reciprocity between the child and caregiver, while responsive feeding entails the caregiver acknowledging the child’s cues of hunger and satiety [33]. Maternal depressive symptoms, anxiety and stress have been implicated in less responsive feeding practices [3] which, in particular circumstances, may lead to child under-nutrition. Alternatively, maternal depressive symptoms may affect children’s stress levels and regulation and may also influence growth. Children who have experienced sensitive, consistent caregiving tend to have more adaptive responses to stress than children exposed to unresponsive or inadequate caregiving [34]. Chronically elevated levels of the stress hormone cortisol are related to lower growth hormone levels in children, possibly resulting in delayed, impaired and even stunted growth [35].

As in most longitudinal studies, loss to follow-up occurred in our study. Due to the study design including all children entering kindergarten, only 20% of the sample was followed at age 6 years. Analyses conducted with and without the 6 year data produced similar estimates of the associations between depressive symptoms and child height and BMI. Mothers who were at higher risk for depressive symptoms (had lower socioeconomic status, were single) were more likely to drop out of the study; they may have experienced more depressive symptoms. Loss of these families, however, is likely to have biased our results towards the null, resulting in conservative estimates of the true association. We also lacked data on parental height and BMI, although we did control for maternal prepregnancy weight and weight gain during pregnancy. The lack of data on children between 9 months and 4 years old limits our ability to model growth during that time period, which may have provided a more nuanced picture of the growth trajectory and the possibility of observing an age by maternal depressive symptoms interaction during this period.

The use of longitudinal growth trajectory analyses is a major study contribution, as it enabled us to observe if maternal depressive symptoms had the same association with child growth throughout the pre-school years until age 6. The CES-D is a well-known and validated measure of depressive symptoms. The ECLS-B provides nationally representative data from a large US cohort study, including a wide range of covariates. Another advantage was that the anthropometric measurements were directly measured at multiple time points.

## Conclusions

In summary, using trajectory analyses of longitudinal data from a nationally representative US sample, we found sustained associations between early maternal depressive symptoms and reduced stature of children at ages 5 and 6. These results have important implications for understanding the long-term effects of maternal depressive symptoms during the first year postpartum on children’s height as late as kindergarten. A better understanding of mechanisms is needed, including the role of caregiving and feeding practices, to explain these relations and why they extend into preschool and kindergarten years. Future studies should include more frequent follow-up points for both maternal depressive symptoms and child growth measures over a longer time period.

### Consent

Written informed consent was obtained from the patient’s guardian/parent/next of kin for the publication of this report and any accompanying images.

## Abbreviations

BMI: Body mass index; CES-D: Center for Epidemiologic Studies Depression Scale; ECLS-B: Early childhood longitudinal program-birth cohort; SD: Standard deviation.

## Competing interests

The authors declare that they have no competing interests.

## Authors’ contributions

PS was instrumental in conceptualizing and designing the study, interpreted results, and wrote the first draft of the manuscript, taking the lead on drafting the introduction, results and discussion sections and in making revisions to the manuscript. AE and RH conducted data analyses, drafted methods section and results tables, and reviewed draft and final versions of the manuscript. SA provided significant input into the statistical analyses and methods of the paper, interpretation of results, and edited the final version to be published. DS and CM provided substantial contributions to the conceptualization and methods of the study, interpretation of the data, and revisions to draft and final versions of the manuscript. All authors read and approved the final manuscript.

## Authors’ information

Dr. Surkan is an Assistant Professor in the Departments of International Health, Population, Family and Reproductive Health (PFRH), and Health Behavior and Society. Her work focuses on social and behavioral determinants of child health outcomes, with a particular emphasis on maternal mental health and child growth and development. In a cross-sectional study of low-income urban families in Brazil, Dr. Surkan found that caregivers’ depressive symptoms are associated with almost a two-fold higher odds of short stature in children ages 6–24 months.

Dr. Ettinger, PhD, MPH, MSW, a recent graduate focusing on early child health and development in the department of PFRH, has conducted longitudinal analyses on trajectories of parenting behaviors in relation to child overweight/obesity as well as extensive analyses using large, nationally representative datasets.

Dr. Hock, PhD, is a Research Fellow in Psychiatry in the Division of Global Psychiatry at Massachusetts General Hopkins Harvard Medical School.

Dr. Ahmed, MBBS, PhD, an Associate Professor in PFRH, is a demographer with statistical and epidemiological expertise as well as a physician who has conducted extensive work on maternal and newborn health in developing countries and has worked extensively modeling growth trajectories and with the ECLS-B data set.

Dr. Strobino, Professor in PFRH, is an expert in maternal and child health and has published widely on topics related to pregnancy, child development and care for women. She was PI on a study of maternal depressive symptoms and children’s growth to age two that shows a negative relation of these symptoms with length-for-age among middle and low income families.

Dr. Minkovitz, Professor in PFRH and Pediatrics at Johns Hopkins University, directs the Women’s and Children’s Health Policy Center. Her recent projects include studies of the effect of father involvement on maternal parenting practices related to obesity and several studies related to maternal depression.

## Pre-publication history

The pre-publication history for this paper can be accessed here:

http://www.biomedcentral.com/1471-2431/14/185/prepub

## Supplementary Material

Additional file 1: Table S1 Partially Adjusted Models of Unweighted Baseline Covariates at 9 Months to Child Height and BMI up to 6 Years^a^. Click here for file
