# Supplementary material for: Early maternal depressive symptoms and child growth trajectories: a longitudinal analysis of a nationally representative US birth cohort
Source: BMC Pediatr. 2014 Jul 21;14:185. doi: 10.1186/1471-2431-14-185 (PMC4114872; doi:10.1186/1471-2431-14-185)
Supplement: Additional file 1: Table S1 — Partially Adjusted Models of Unweighted Baseline Covariates at 9 Months to Child Height and BMI up to 6 Yearsa. [file 1471-2431-14-185-S1.docx]

**Additional file 1: Table S1.** Partially Adjusted Models of Unweighted Baseline Covariates at 9 Months to Child Height and BMI up to 6 Years^a^

|  | **N (%) or Mean (SD)** | **Height (cm)**  ***N*=6550** | **Body Mass Index (kg/m^2^)**  ***N*=6550** |
| --- | --- | --- | --- |
|  |  | **β (95% CI)** | **β (95% CI)** |
| **Household income**  > $25,000  $25,000 - $49,999  $50,000- $99,999  ≤ $100,000 | 2300 (35.3)  1900 (29.2)  1600 (24.7)  700 (10.8) | Reference  0.16 (-0.05, 0.37)  0.22 (0.003, 0.44)*  0.67 (0.38, 0.97)*** | Reference  -0.20 (-0.32, -0.08)**  -0.44 (-0.55, -0.30)***  -0.34 (-0.50, -0.18)*** |
| **Household food security**  Food insecure  Food secure | 800 (12.1)  5750 (87.9) | Reference  0.14 (-0.12, 0.39) | Reference  -0.21 (-0.35, -0.06)** |
| **Home ownership**  Do not own home  Own your home | 3500 (53.2)  3050 (46.8) | Reference  0.01 (-0.16, 0.18) | Reference  -0.31 (-0.41, -0.22)*** |
| **Family structure**  Single parent  Two parents | 1400 (21.2)  5150 (78.9) | Reference  0.01 (-0.20, 0.21) | Reference  -0.25 (-0.36, -0.13)*** |
| **Age (years)**  15-19  20-24  25-29  30-34  ≥35 | 500 (7.7)  1650 (25.2)  1600 (24.5)  1600 (24.2)  1200 (18.5) | Reference  -0.07 (-0.42, 0.28)  0.10 (-0.25, 0.44)  0.10 (-0.25, 0.44)  -0.01 (-0.38, 0.34) | Reference  -0.15 (-0.34, 0.05)  -0.05 (-0.24, 0.15)  -0.31 (-0.50, -0.11)**  -0.24 (-0.44, -0.04)* |
| **Race/ethnicity**  White, non-Hispanic  Black, non-Hispanic  Asian, non-Hispanic  Hispanic  Other | 3050 (46.3)  1000 (15.5)  850 (12.7)  1100 (17.0)  550 (8.6) | Reference  -0.25 (-0.49, -0.002)*  0.36 (0.10, 0.63)**  0.23 (-0.01, 0.47)  0.70 (0.39, 1.00)*** | Reference  0.07 (-0.07, 0.20)  -0.34 (-0.49, -0.20)***  0.34 (0.22, 0.48)***  0.42 (0.24, 0.59)*** |
| **Education**  Some HS or less  HS graduate  Some college  College and above | 1150 (17.6)  2000 (30.4)  1650 (25.2)  1750 (26.9) | Reference  0.27 (0.02, 0.52)*  0.39 (0.13, 0.65)**  0.55 (0.29, 0.80)*** | Reference  -0.05 (-0.19, 0.09)  -0.23 (-0.37, -0.08)***  -0.45 (-0.59, -0.31)*** |
| **Employment**  Not working  Working | 2550 (39.2)  4000 (60.8) | Reference  0.25 (0.08, 0.43)** | Reference  0.15 (0.06, 0.25)*** |
| **Pre-pregnancy weight**  Low tertile  Middle tertile  High tertile | 2200 (33.8)  2200 (33.6)  2150 (32.6) | Reference  0.60 (0.39, 0.80)***  0.77 (0.57, 0.98)*** | Reference  0.31 (0.20, 0.42)***  0.73 (0.62, 0.85)*** |
| **Pregnancy weight gain**  Low tertile  Middle tertile  High tertile | 2400 (36.8)  2000 (29.9)  2200 (33.3) | Reference  0.62 (0.41, 0.82)***  1.10 (0.90, 1.30)*** | Reference  0.11 (-0.003, 0.25)^  0.39 (0.27, 0.50)*** |
| **Parity/First-born**  Later birth  First birth | 4400 (66.9)  2150 (33.1) | Reference  0.11 (-0.07, 0.28) | Reference  0.02 (-0.08, 0.12) |
| **Maternal Smoking^b^**  Never smoked  Smoked after birth  Smoked during and after birth | 4800 (73.0)  300 (4.6)  1450 (22.4) | Reference  0.12 (-0.29, 0.52)  -0.36 (-0.57, -0.16)*** | Reference  0.45 (0.22, 0.68)***  0.28 (0.17, 0.39)*** |
| **Health Status**  Good/fair/poor  Excellent/very good | 850 (12.8)  5700 (87.2) | Reference  1.32 (1.07, 1.57)*** | Reference  0.20 (0.06, 0.35)* |
| **Birthweight**  > 2500 g  1500-2500 g  ≤ 1500 g | 5300 (81.0)  650 (10.1)  600 (8.9) | Reference  -2.43 (-2.69, -2.18)***  -4.81 (-5.08, -4.54)*** | Reference  -0.45 (-0.61, -0.30)***  -0.78 (-0.98, -0.61)*** |
| **Sex^c^**  Girl  Boy | 3200 (48.9)  3340 (51.0) | Reference  1.38 (1.21, 1.55)*** | Reference  0.38 (0.29, 0.48)*** |
| **Breast fed**  Never  Ever | 2000 (30.7)  4550 (69.3) | Reference  0.17 (-0.01, 0.35)^ | Reference  -0.33 (-0.43, -0.23)*** |
| **Gestational age** (Mean, SD) | 37.9 (3.7) | 0.34 (0.32, 0.36)*** | 0.05 (0.04, 0.06)*** |

^a^ Unweighted random effects (intercept and slope) models adjusted for child age, child age^2^, and child sex

^b^ No mothers smoked only during and not after pregnancy.

^c^ Adjusted for child age only.

* p < 0.05. ** p < 0.01. ***p < 0.001.

^ p < 0.10.
